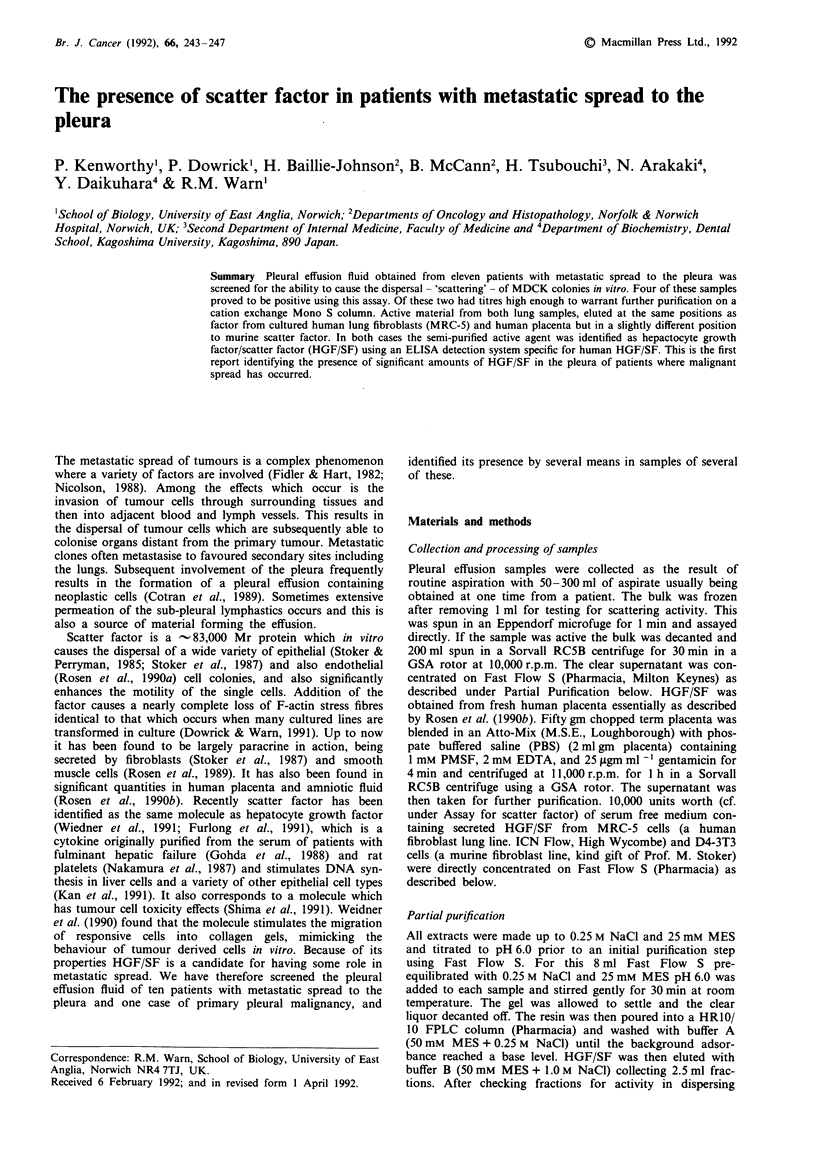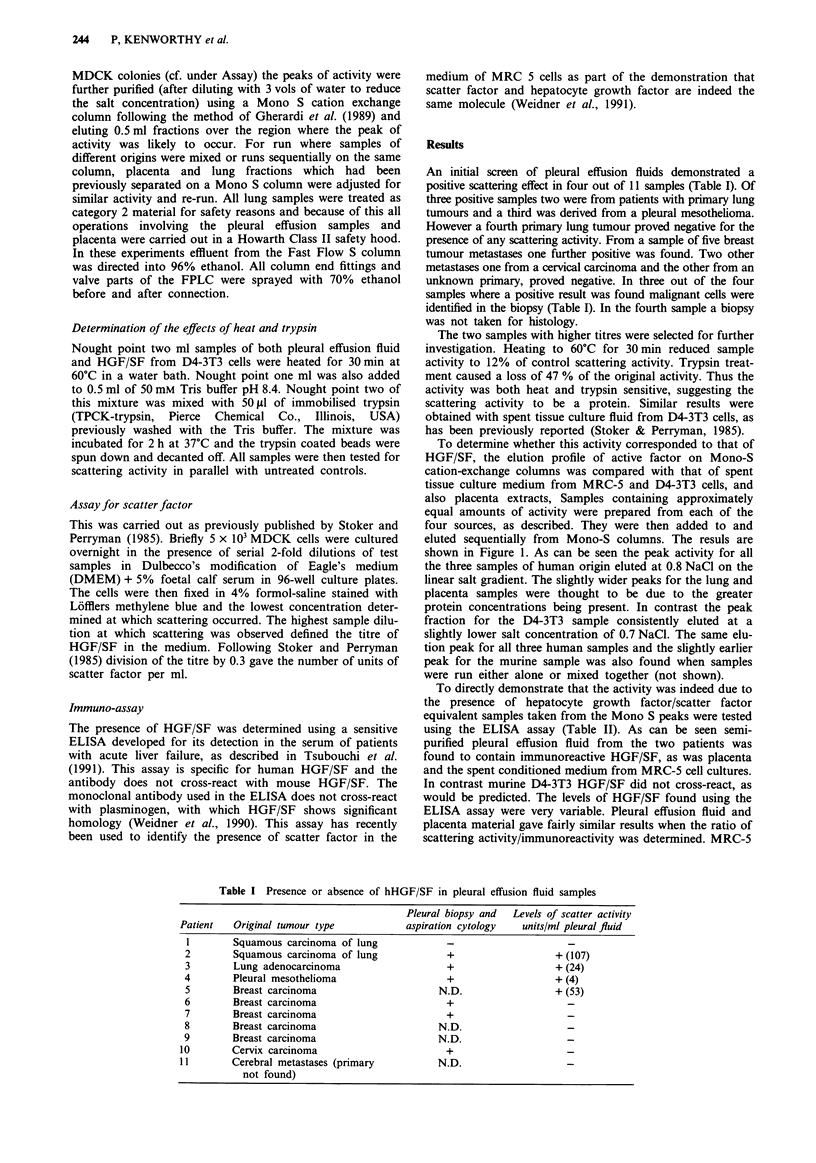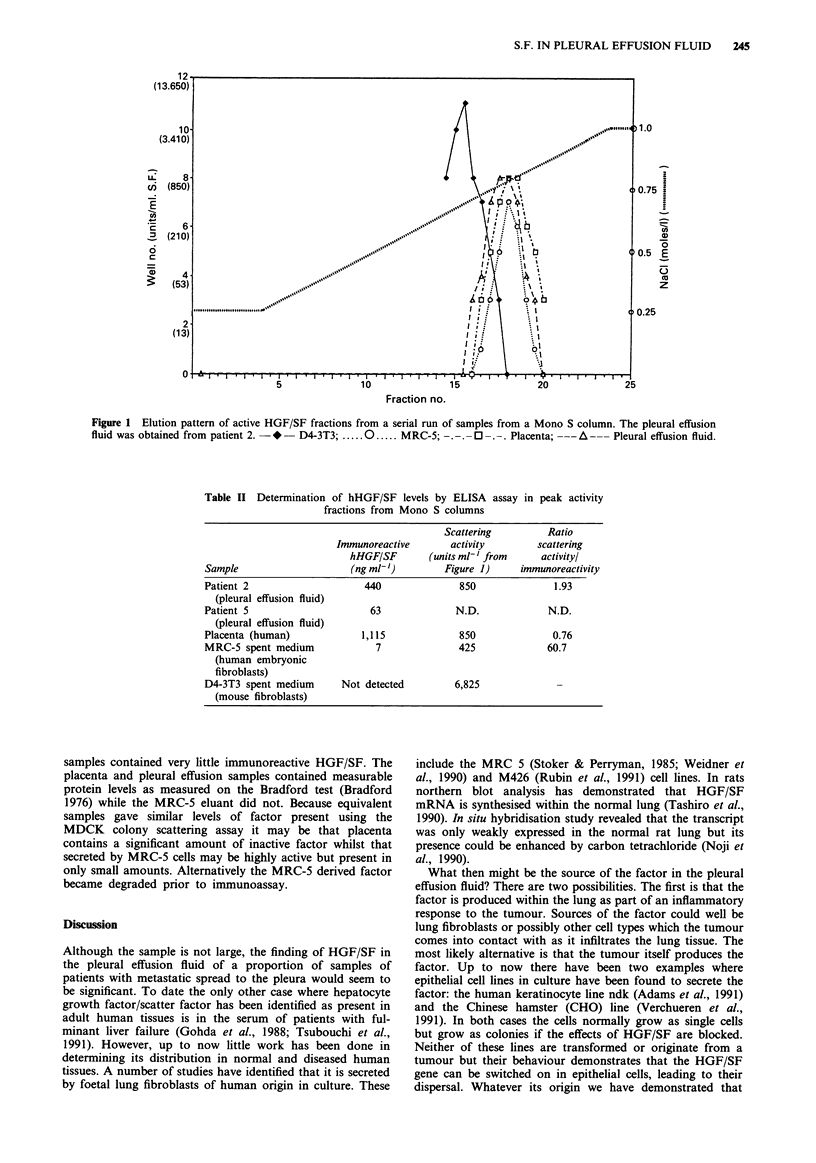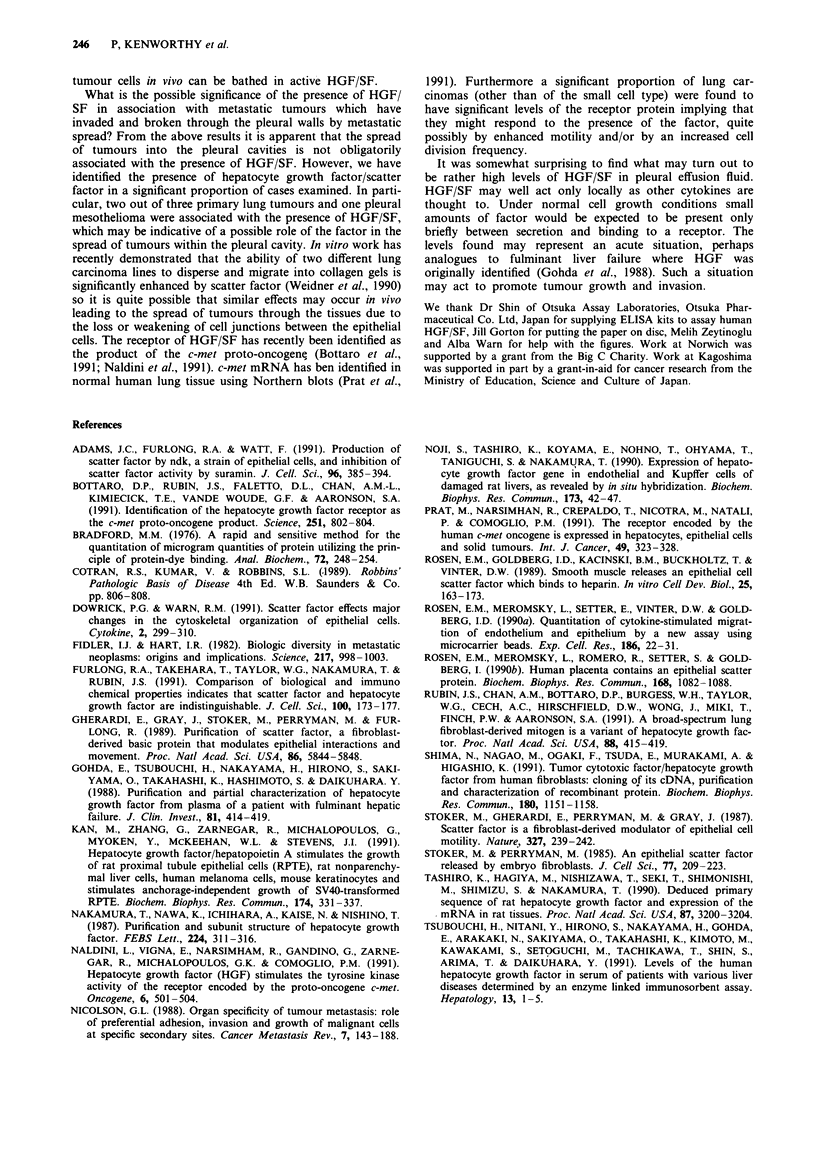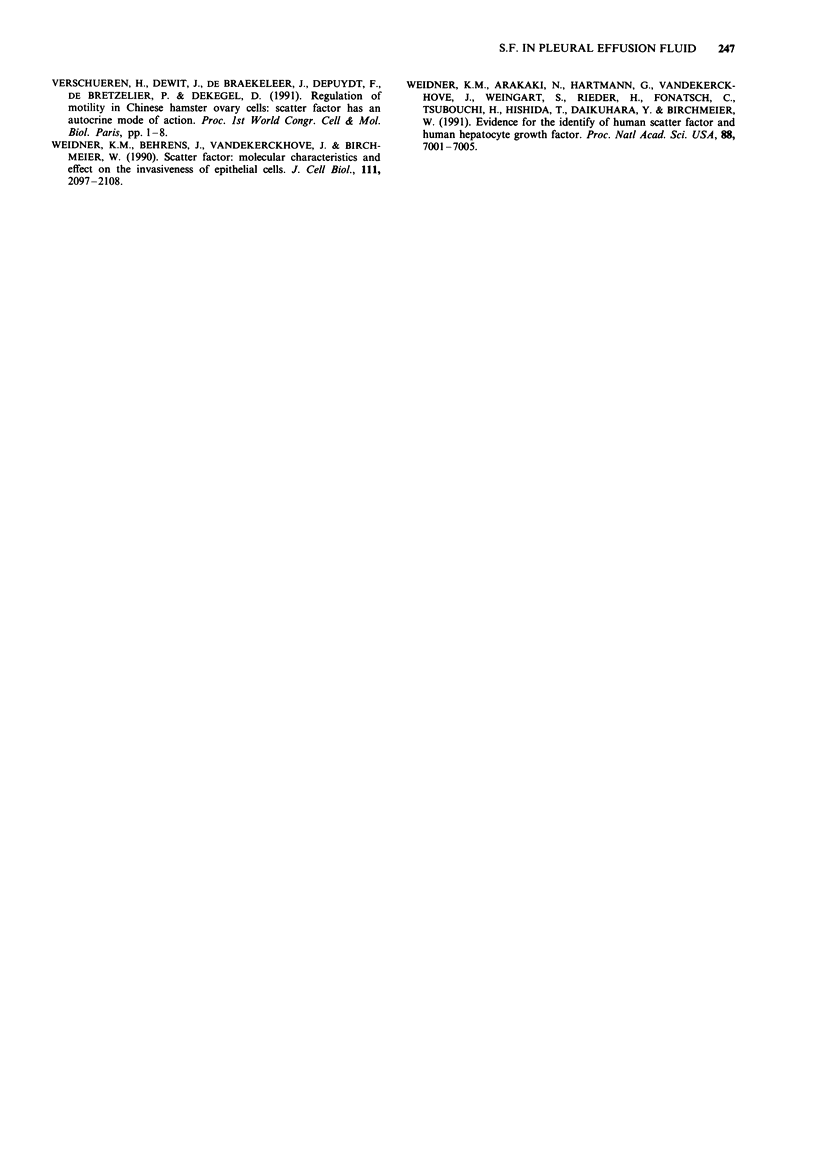# The presence of scatter factor in patients with metastatic spread to the pleura.

**DOI:** 10.1038/bjc.1992.251

**Published:** 1992-08

**Authors:** P. Kenworthy, P. Dowrick, H. Baillie-Johnson, B. McCann, H. Tsubouchi, N. Arakaki, Y. Daikuhara, R. M. Warn

**Affiliations:** School of Biology, University of East Anglia, Norwich, UK.

## Abstract

Pleural effusion fluid obtained from eleven patients with metastatic spread to the pleura was screened for the ability to cause the dispersal--'scattering'--of MDCK colonies in vitro. Four of these samples proved to be positive using this assay. Of these two had titres high enough to warrant further purification on a cation exchange Mono S column. Active material from both lung samples, eluted at the same positions as factor from cultured human lung fibroblasts (MRC-5) and human placenta but in a slightly different position to murine scatter factor. In both cases the semi-purified active agent was identified as hepatocyte growth factor/scatter factor (HGF/SF) using an ELISA detection system specific for human HGF/SF. This is the first report identifying the presence of significant amounts of HGF/SF in the pleura of patients where malignant spread has occurred.


Br. J. Cancer (1992), 66, 243–247 © Macmillan Press Ltd., 1992

### The presence of scatter factor in patients with metastatic spread to the pleura

P. Kenworthy[1], P. Dowrick[1], H. Baillie-Johnson[2], B. McCann[2], H. Tsubouchi[3], N. Arakaki[4], Y. Daikuhara[4] & R.M. Warn[1]

[1]*School of Biology, University of East Anglia, Norwich;* [2]*Departments of Oncology and Histopathology, Norfolk & Norwich Hospital, Norwich, UK;* [3]*Second Department of Internal Medicine, Faculty of Medicine and* [4]*Department of Biochemistry, Dental School, Kagoshima University, Kagoshima, 890 Japan.*

**Summary** Pleural effusion fluid obtained from eleven patients with metastatic spread to the pleura was screened for the ability to cause the dispersal – 'scattering' – of MDCK colonies *in vitro*. Four of these samples proved to be positive using this assay. Of these two had titres high enough to warrant further purification on a cation exchange Mono S column. Active material from both lung samples, eluted at the same positions as factor from cultured human lung fibroblasts (MRC-5) and human placenta but in a slightly different position to murine scatter factor. In both cases the semi-purified active agent was identified as hepactocyte growth factor/scatter factor (HGF/SF) using an ELISA detection system specific for human HGF/SF. This is the first report identifying the presence of significant amounts of HGF/SF in the pleura of patients where malignant spread has occurred.

The metastatic spread of tumours is a complex phenomenon where a variety of factors are involved (Fidler & Hart, 1982; Nicolson, 1988). Among the effects which occur is the invasion of tumour cells through surrounding tissues and then into adjacent blood and lymph vessels. This results in the dispersal of tumour cells which are subsequently able to colonise organs distant from the primary tumour. Metastatic clones often metastasise to favoured secondary sites including the lungs. Subsequent involvement of the pleura frequently results in the formation of a pleural effusion containing neoplastic cells (Cotran *et al.*, 1989). Sometimes extensive permeation of the sub-pleural lymphastics occurs and this is also a source of material forming the effusion.

Scatter factor is a ~83,000 Mr protein which *in vitro* causes the dispersal of a wide variety of epithelial (Stoker & Perryman, 1985; Stoker *et al.*, 1987) and also endothelial (Rosen *et al.*, 1990*a*) cell colonies, and also significantly enhances the motility of the single cells. Addition of the factor causes a nearly complete loss of F-actin stress fibres identical to that which occurs when many cultured lines are transformed in culture (Dowrick & Warn, 1991). Up to now it has been found to be largely paracrine in action, being secreted by fibroblasts (Stoker *et al.*, 1987) and smooth muscle cells (Rosen *et al.*, 1989). It has also been found in significant quantities in human placenta and amniotic fluid (Rosen *et al.*, 1990*b*). Recently scatter factor has been identified as the same molecule as hepatocyte growth factor (Wiedner *et al.*, 1991; Furlong *et al.*, 1991), which is a cytokine originally purified from the serum of patients with fulminant hepatic failure (Gohda *et al.*, 1988) and rat platelets (Nakamura *et al.*, 1987) and stimulates DNA synthesis in liver cells and a variety of other epithelial cell types (Kan *et al.*, 1991). It also corresponds to a molecule which has tumour cell toxicity effects (Shima *et al.*, 1991). Weidner *et al.* (1990) found that the molecule stimulates the migration of responsive cells into collagen gels, mimicking the behaviour of tumour derived cells *in vitro*. Because of its properties HGF/SF is a candidate for having some role in metastatic spread. We have therefore screened the pleural effusion fluid of ten patients with metastatic spread to the pleura and one case of primary pleural malignancy, and identified its presence by several means in samples of several of these.

Correspondence: R.M. Warn, School of Biology, University of East Anglia, Norwich NR4 7TJ, UK.
Received 6 February 1992; and in revised form 1 April 1992.

### Materials and methods

### *Collection and processing of samples*

Pleural effusion samples were collected as the result of routine aspiration with 50–300 ml of aspirate usually being obtained at one time from a patient. The bulk was frozen after removing 1 ml for testing for scattering activity. This was spun in an Eppendorf microfuge for 1 min and assayed directly. If the sample was active the bulk was decanted and 200 ml spun in a Sorvall RC5B centrifuge for 30 min in a GSA rotor at 10,000 r.p.m. The clear supernatant was concentrated on Fast Flow S (Pharmacia, Milton Keynes) as described under Partial Purification below. HGF/SF was obtained from fresh human placenta essentially as described by Rosen *et al.* (1990*b*). Fifty gm chopped term placenta was blended in an Atto-Mix (M.S.E., Loughborough) with phospate buffered saline (PBS) (2 ml gm placenta) containing 1 mM PMSF, 2 mM EDTA, and 25 $\mu$gm ml $^{-1}$ gentamicin for 4 min and centrifuged at 11,000 r.p.m. for 1 h in a Sorvall RC5B centrifuge using a GSA rotor. The supernatant was then taken for further purification. 10,000 units worth (cf. under Assay for scatter factor) of serum free medium containing secreted HGF/SF from MRC-5 cells (a human fibroblast lung line. ICN Flow, High Wycombe) and D4-3T3 cells (a murine fibroblast line, kind gift of Prof. M. Stoker) were directly concentrated on Fast Flow S (Pharmacia) as described below.

### *Partial purification*

All extracts were made up to 0.25 M NaCl and 25 mM MES and titrated to pH 6.0 prior to an initial purification step using Fast Flow S. For this 8 ml Fast Flow S pre-equilibrated with 0.25 M NaCl and 25 mM MES pH 6.0 was added to each sample and stirred gently for 30 min at room temperature. The gel was allowed to settle and the clear liquor decanted off. The resin was then poured into a HR10/10 FPLC column (Pharmacia) and washed with buffer A (50 mM MES + 0.25 M NaCl) until the background adsorbance reached a base level. HGF/SF was then eluted with buffer B (50 mM MES + 1.0 M NaCl) collecting 2.5 ml fractions. After checking fractions for activity in dispersing

MDCK colonies (cf. under Assay) the peaks of activity were further purified (after diluting with 3 vols of water to reduce the salt concentration) using a Mono S cation exchange column following the method of Gherardi et al. (1989) and eluting 0.5 ml fractions over the region where the peak of activity was likely to occur. For run where samples of different origins were mixed or runs sequentially on the same column, placenta and lung fractions which had been previously separated on a Mono S column were adjusted for similar activity and re-run. All lung samples were treated as category 2 material for safety reasons and because of this all operations involving the pleural effusion samples and placenta were carried out in a Howarth Class II safety hood. In these experiments effluent from the Fast Flow S column was directed into 96% ethanol. All column end fittings and valve parts of the FPLC were sprayed with 70% ethanol before and after connection.

*Determination of the effects of heat and trypsin*

Nought point two ml samples of both pleural effusion fluid and HGF/SF from D4-3T3 cells were heated for 30 min at 60°C in a water bath. Nought point one ml was also added to 0.5 ml of 50 mM Tris buffer pH 8.4. Nought point two of this mixture was mixed with 50 µl of immobilised trypsin (TPCK-trypsin, Pierce Chemical Co., Illinois, USA) previously washed with the Tris buffer. The mixture was incubated for 2 h at 37°C and the trypsin coated beads were spun down and decanted off. All samples were then tested for scattering activity in parallel with untreated controls.

*Assay for scatter factor*

This was carried out as previously published by Stoker and Perryman (1985). Briefly $5 \times 10^3$ MDCK cells were cultured overnight in the presence of serial 2-fold dilutions of test samples in Dulbecco's modification of Eagle's medium (DMEM) + 5% foetal calf serum in 96-well culture plates. The cells were then fixed in 4% formol-saline stained with Löfflers methylene blue and the lowest concentration determined at which scattering occurred. The highest sample dilution at which scattering was observed defined the titre of HGF/SF in the medium. Following Stoker and Perryman (1985) division of the titre by 0.3 gave the number of units of scatter factor per ml.

*Immuno-assay*

The presence of HGF/SF was determined using a sensitive ELISA developed for its detection in the serum of patients with acute liver failure, as described in Tsubouchi et al. (1991). This assay is specific for human HGF/SF and the antibody does not cross-react with mouse HGF/SF. The monoclonal antibody used in the ELISA does not cross-react with plasminogen, with which HGF/SF shows significant homology (Weidner et al., 1990). This assay has recently been used to identify the presence of scatter factor in the medium of MRC 5 cells as part of the demonstration that scatter factor and hepatocyte growth factor are indeed the same molecule (Weidner et al., 1991).

### Results

An initial screen of pleural effusion fluids demonstrated a positive scattering effect in four out of 11 samples (Table I). Of three positive samples two were from patients with primary lung tumours and a third was derived from a pleural mesothelioma. However a fourth primary lung tumour proved negative for the presence of any scattering activity. From a sample of five breast tumour metastases one further positive was found. Two other metastases one from a cervical carcinoma and the other from an unknown primary, proved negative. In three out of the four samples where a positive result was found malignant cells were identified in the biopsy (Table I). In the fourth sample a biopsy was not taken for histology.

The two samples with higher titres were selected for further investigation. Heating to 60°C for 30 min reduced sample activity to 12% of control scattering activity. Trypsin treatment caused a loss of 47 % of the original activity. Thus the activity was both heat and trypsin sensitive, suggesting the scattering activity to be a protein. Similar results were obtained with spent tissue culture fluid from D4-3T3 cells, as has been previously reported (Stoker & Perryman, 1985).

To determine whether this activity corresponded to that of HGF/SF, the elution profile of active factor on Mono-S cation-exchange columns was compared with that of spent tissue culture medium from MRC-5 and D4-3T3 cells, and also placenta extracts, Samples containing approximately equal amounts of activity were prepared from each of the four sources, as described. They were then added to and eluted sequentially from Mono-S columns. The resuls are shown in Figure 1. As can be seen the peak activity for all the three samples of human origin eluted at 0.8 NaCl on the linear salt gradient. The slightly wider peaks for the lung and placenta samples were thought to be due to the greater protein concentrations being present. In contrast the peak fraction for the D4-3T3 sample consistently eluted at a slightly lower salt concentration of 0.7 NaCl. The same elution peak for all three human samples and the slightly earlier peak for the murine sample was also found when samples were run either alone or mixed together (not shown).

To directly demonstrate that the activity was indeed due to the presence of hepatocyte growth factor/scatter factor equivalent samples taken from the Mono S peaks were tested using the ELISA assay (Table II). As can be seen semi-purified pleural effusion fluid from the two patients was found to contain immunoreactive HGF/SF, as was placenta and the spent conditioned medium from MRC-5 cell cultures. In contrast murine D4-3T3 HGF/SF did not cross-react, as would be predicted. The levels of HGF/SF found using the ELISA assay were very variable. Pleural effusion fluid and placenta material gave fairly similar results when the ratio of scattering activity/immunoreactivity was determined. MRC-5

**Table I** Presence or absence of hHGF/SF in pleural effusion fluid samples

| *Patient* | *Original tumour type* | *Pleural biopsy and aspiration cytology* | *Levels of scatter activity units/ml pleural fluid* |
|---|---|---|---|
| 1 | Squamous carcinoma of lung | – | – |
| 2 | Squamous carcinoma of lung | + | + (107) |
| 3 | Lung adenocarcinoma | + | + (24) |
| 4 | Pleural mesothelioma | + | + (4) |
| 5 | Breast carcinoma | N.D. | + (53) |
| 6 | Breast carcinoma | + | – |
| 7 | Breast carcinoma | + | – |
| 8 | Breast carcinoma | N.D. | – |
| 9 | Breast carcinoma | N.D. | – |
| 10 | Cervix carcinoma | + | – |
| 11 | Cerebral metastases (primary not found) | N.D. | – |

**Figure 1** Elution pattern of active HGF/SF fractions from a serial run of samples from a Mono S column. The pleural effusion fluid was obtained from patient 2. —◆— D4-3T3; .....O..... MRC-5; -.-.-□-.-. Placenta; ---△--- Pleural effusion fluid.

**Table II** Determination of hHGF/SF levels by ELISA assay in peak activity fractions from Mono S columns

| *Sample* | *Immunoreactive hHGF/SF (ng ml⁻¹)* | *Scattering activity (units ml⁻¹ from Figure 1)* | *Ratio scattering activity/ immunoreactivity* |
|---|---|---|---|
| Patient 2 (pleural effusion fluid) | 440 | 850 | 1.93 |
| Patient 5 (pleural effusion fluid) | 63 | N.D. | N.D. |
| Placenta (human) | 1,115 | 850 | 0.76 |
| MRC-5 spent medium (human embryonic fibroblasts) | 7 | 425 | 60.7 |
| D4-3T3 spent medium (mouse fibroblasts) | Not detected | 6,825 | – |

samples contained very little immunoreactive HGF/SF. The placenta and pleural effusion samples contained measurable protein levels as measured on the Bradford test (Bradford 1976) while the MRC-5 eluant did not. Because equivalent samples gave similar levels of factor present using the MDCK colony scattering assay it may be that placenta contains a significant amount of inactive factor whilst that secreted by MRC-5 cells may be highly active but present in only small amounts. Alternatively the MRC-5 derived factor became degraded prior to immunoassay.

### Discussion

Although the sample is not large, the finding of HGF/SF in the pleural effusion fluid of a proportion of samples of patients with metastatic spread to the pleura would seem to be significant. To date the only other case where hepatocyte growth factor/scatter factor has been identified as present in adult human tissues is in the serum of patients with fulminant liver failure (Gohda *et al.*, 1988; Tsubouchi *et al.*, 1991). However, up to now little work has been done in determining its distribution in normal and diseased human tissues. A number of studies have identified that it is secreted by foetal lung fibroblasts of human origin in culture. These include the MRC 5 (Stoker & Perryman, 1985; Weidner *et al.*, 1990) and M426 (Rubin *et al.*, 1991) cell lines. In rats northern blot analysis has demonstrated that HGF/SF mRNA is synthesised within the normal lung (Tashiro *et al.*, 1990). *In situ* hybridisation study revealed that the transcript was only weakly expressed in the normal rat lung but its presence could be enhanced by carbon tetrachloride (Noji *et al.*, 1990).

What then might be the source of the factor in the pleural effusion fluid? There are two possibilities. The first is that the factor is produced within the lung as part of an inflammatory response to the tumour. Sources of the factor could well be lung fibroblasts or possibly other cell types which the tumour comes into contact with as it infiltrates the lung tissue. The most likely alternative is that the tumour itself produces the factor. Up to now there have been two examples where epithelial cell lines in culture have been found to secrete the factor: the human keratinocyte line ndk (Adams *et al.*, 1991) and the Chinese hamster (CHO) line (Verchueren *et al.*, 1991). In both cases the cells normally grow as single cells but grow as colonies if the effects of HGF/SF are blocked. Neither of these lines are transformed or originate from a tumour but their behaviour demonstrates that the HGF/SF gene can be switched on in epithelial cells, leading to their dispersal. Whatever its origin we have demonstrated that tumour cells *in vivo* can be bathed in active HGF/SF.

What is the possible significance of the presence of HGF/SF in association with metastatic tumours which have invaded and broken through the pleural walls by metastatic spread? From the above results it is apparent that the spread of tumours into the pleural cavities is not obligatorily associated with the presence of HGF/SF. However, we have identified the presence of hepatocyte growth factor/scatter factor in a significant proportion of cases examined. In particular, two out of three primary lung tumours and one pleural mesothelioma were associated with the presence of HGF/SF, which may be indicative of a possible role of the factor in the spread of tumours within the pleural cavity. *In vitro* work has recently demonstrated that the ability of two different lung carcinoma lines to disperse and migrate into collagen gels is significantly enhanced by scatter factor (Weidner *et al.*, 1990) so it is quite possible that similar effects may occur *in vivo* leading to the spread of tumours through the tissues due to the loss or weakening of cell junctions between the epithelial cells. The receptor of HGF/SF has recently been identified as the product of the *c-met* proto-oncogene (Bottaro *et al.*, 1991; Naldini *et al.*, 1991). *c-met* mRNA has ben identified in normal human lung tissue using Northern blots (Prat *et al.*, 1991). Furthermore a significant proportion of lung carcinomas (other than of the small cell type) were found to have significant levels of the receptor protein implying that they might respond to the presence of the factor, quite possibly by enhanced motility and/or by an increased cell division frequency.

It was somewhat surprising to find what may turn out to be rather high levels of HGF/SF in pleural effusion fluid. HGF/SF may well act only locally as other cytokines are thought to. Under normal cell growth conditions small amounts of factor would be expected to be present only briefly between secretion and binding to a receptor. The levels found may represent an acute situation, perhaps analogues to fulminant liver failure where HGF was originally identified (Gohda *et al.*, 1988). Such a situation may act to promote tumour growth and invasion.

We thank Dr Shin of Otsuka Assay Laboratories, Otsuka Pharmaceutical Co. Ltd, Japan for supplying ELISA kits to assay human HGF/SF, Jill Gorton for putting the paper on disc, Melih Zeytinoglu and Alba Warn for help with the figures. Work at Norwich was supported by a grant from the Big C Charity. Work at Kagoshima was supported in part by a grant-in-aid for cancer research from the Ministry of Education, Science and Culture of Japan.

### References

ADAMS, J.C., FURLONG, R.A. & WATT, F. (1991). Production of scatter factor by ndk, a strain of epithelial cells, and inhibition of scatter factor activity by suramin. *J. Cell. Sci.*, **96**, 385–394.

BOTTARO, D.P., RUBIN, J.S., FALETTO, D.L., CHAN, A.M.-L., KIMIECICK, T.E., VANDE WOUDE, G.F. & AARONSON, S.A. (1991). Identification of the hepatocyte growth factor receptor as the *c-met* proto-oncogene product. *Science*, **251**, 802–804.

BRADFORD, M.M. (1976). A rapid and sensitive method for the quantitation of microgram quantities of protein utilizing the principle of protein-dye binding. *Anal. Biochem.*, **72**, 248–254.

COTRAN, R.S., KUMAR, V. & ROBBINS, S.L. (1989). *Robbins' Pathologic Basis of Disease* 4th Ed. W.B. Saunders & Co. pp. 806–808.

DOWRICK, P.G. & WARN, R.M. (1991). Scatter factor effects major changes in the cytoskeletal organization of epithelial cells. *Cytokine*, **2**, 299–310.

FIDLER, I.J. & HART, I.R. (1982). Biologic diversity in metastatic neoplasms: origins and implications. *Science*, **217**, 998–1003.

FURLONG, R.A., TAKEHARA, T., TAYLOR, W.G., NAKAMURA, T. & RUBIN, J.S. (1991). Comparison of biological and immuno chemical properties indicates that scatter factor and hepatocyte growth factor are indistinguishable. *J. Cell. Sci.*, **100**, 173–177.

GHERARDI, E., GRAY, J., STOKER, M., PERRYMAN, M. & FURLONG, R. (1989). Purification of scatter factor, a fibroblast-derived basic protein that modulates epithelial interactions and movement. *Proc. Natl Acad. Sci. USA*, **86**, 5844–5848.

GOHDA, E., TSUBOUCHI, H., NAKAYAMA, H., HIRONO, S., SAKIYAMA, O., TAKAHASHI, K., HASHIMOTO, S. & DAIKUHARA. Y. (1988). Purification and partial characterization of hepatocyte growth factor from plasma of a patient with fulminant hepatic failure. *J. Clin. Invest.*, **81**, 414–419.

KAN, M., ZHANG, G., ZARNEGAR, R., MICHALOPOULOS, G., MYOKEN, Y., MCKEEHAN, W.L. & STEVENS, J.I. (1991). Hepatocyte growth factor/hepatopoietin A stimulates the growth of rat proximal tubule epithelial cells (RPTE), rat nonparenchymal liver cells, human melanoma cells, mouse keratinocytes and stimulates anchorage-independent growth of SV40-transformed RPTE. *Biochem. Biophys. Res. Commun.*, **174**, 331–337.

NAKAMURA, T., NAWA, K., ICHIHARA, A., KAISE, N. & NISHINO, T. (1987). Purification and subunit structure of hepatocyte growth factor. *FEBS Lett.*, **224**, 311–316.

NALDINI, L., VIGNA, E., NARSIMHAM, R., GANDINO, G., ZARNEGAR, R., MICHALOPOULOS, G.K. & COMOGLIO, P.M. (1991). Hepatocyte growth factor (HGF) stimulates the tyrosine kinase activity of the receptor encoded by the proto-oncogene *c-met*. *Oncogene*, **6**, 501–504.

NICOLSON, G.L. (1988). Organ specificity of tumour metastasis: role of preferential adhesion, invasion and growth of malignant cells at specific secondary sites. *Cancer Metastasis Rev.*, **7**, 143–188.

NOJI, S., TASHIRO, K., KOYAMA, E., NOHNO, T., OHYAMA, T., TANIGUCHI, S. & NAKAMURA, T. (1990). Expression of hepatocyte growth factor gene in endothelial and Kupffer cells of damaged rat livers, as revealed by *in situ* hybridization. *Biochem. Biophys. Res. Commun.*, **173**, 42–47.

PRAT, M., NARSIMHAN, R., CREPALDO, T., NICOTRA, M., NATALI, P. & COMOGLIO, P.M. (1991). The receptor encoded by the human *c-met* oncogene is expressed in hepatocytes, epithelial cells and solid tumours. *Int. J. Cancer*, **49**, 323–328.

ROSEN, E.M., GOLDBERG, I.D., KACINSKI, B.M., BUCKHOLTZ, T. & VINTER, D.W. (1989). Smooth muscle releases an epithelial cell scatter factor which binds to heparin. *In vitro Cell Dev. Biol.*, **25**, 163–173.

ROSEN, E.M., MEROMSKY, L., SETTER, E., VINTER, D.W. & GOLDBERG, I.D. (1990*a*). Quantitation of cytokine-stimulated migration of endothelium and epithelium by a new assay using microcarrier beads. *Exp. Cell. Res.*, **186**, 22–31.

ROSEN, E.M., MEROMSKY, L., ROMERO, R., SETTER, S. & GOLDBERG, I. (1990*b*). Human placenta contains an epithelial scatter protein. *Biochem. Biophys. Res. Commun.*, **168**, 1082–1088.

RUBIN, J.S., CHAN, A.M., BOTTARO, D.P., BURGESS, W.H., TAYLOR, W.G., CECH, A.C., HIRSCHFIELD, D.W., WONG, J., MIKI, T., FINCH, P.W. & AARONSON, S.A. (1991). A broad-spectrum lung fibroblast-derived mitogen is a variant of hepatocyte growth factor. *Proc. Natl Acad. Sci. USA*, **88**, 415–419.

SHIMA, N., NAGAO, M., OGAKI, F., TSUDA, E., MURAKAMI, A. & HIGASHIO, K. (1991). Tumor cytotoxic factor/hepatocyte growth factor from human fibroblasts: cloning of its cDNA, purification and characterization of recombinant protein. *Biochem. Biophys. Res. Commun.*, **180**, 1151–1158.

STOKER, M., GHERARDI, E., PERRYMAN, M. & GRAY, J. (1987). Scatter factor is a fibroblast-derived modulator of epithelial cell motility. *Nature*, **327**, 239–242.

STOKER, M. & PERRYMAN, M. (1985). An epithelial scatter factor released by embryo fibroblasts. *J. Cell Sci.*, **77**, 209–223.

TASHIRO, K., HAGIYA, M., NISHIZAWA, T., SEKI, T., SHIMONISHI, M., SHIMIZU, S. & NAKAMURA, T. (1990). Deduced primary sequence of rat hepatocyte growth factor and expression of the mRNA in rat tissues. *Proc. Natl Acad. Sci. USA*, **87**, 3200–3204.

TSUBOUCHI, H., NITANI, Y., HIRONO, S., NAKAYAMA, H., GOHDA, E., ARAKAKI, N., SAKIYAMA, O., TAKAHASHI, K., KIMOTO, M., KAWAKAMI, S., SETOGUCHI, M., TACHIKAWA, T., SHIN, S., ARIMA, T. & DAIKUHARA, Y. (1991). Levels of the human hepatocyte growth factor in serum of patients with various liver diseases determined by an enzyme linked immunosorbent assay. *Hepatology*, **13**, 1–5.

VERSCHUEREN, H., DEWIT, J., DE BRAEKELEER, J., DEPUYDT, F., DE BRETZELIER, P. & DEKEGEL, D. (1991). Regulation of motility in Chinese hamster ovary cells: scatter factor has an autocrine mode of action. *Proc. 1st World Congr. Cell & Mol. Biol. Paris*, pp. 1–8.

WEIDNER, K.M., BEHRENS, J., VANDEKERCKHOVE, J. & BIRCHMEIER, W. (1990). Scatter factor: molecular characteristics and effect on the invasiveness of epithelial cells. *J. Cell Biol.*, **111**, 2097–2108.

WEIDNER, K.M., ARAKAKI, N., HARTMANN, G., VANDEKERCKHOVE, J., WEINGART, S., RIEDER, H., FONATSCH, C., TSUBOUCHI, H., HISHIDA, T., DAIKUHARA, Y. & BIRCHMEIER, W. (1991). Evidence for the identify of human scatter factor and human hepatocyte growth factor. *Proc. Natl Acad. Sci. USA*, **88**, 7001–7005.